# A Comparison on Some Interval Mapping Approaches for QTL Detection

**DOI:** 10.6026/97320630015090

**Published:** 2019-02-28

**Authors:** Zobaer Akond, Md.Jahangir Alam, Mohammad Nazmol Hasan, Md.Shalim Uddin, Munirul Alam, Md.Nurul Haque Mollah

**Affiliations:** 1 Bioinformatics Lab,Department of Statistics,University of Rajshahi,Rajshahi-6205,Bangladesh; 2 Institute of Environmental Science,University of Rajshahi-6205,Bangladesh; 3 Agricultural Statistics and Information and Communication Technology (ASICT) Division,Bangladesh Agricultural Research Institute (BARI),Joydebpur,Gazipur-1701,Bangladesh;; 4 Emerging Infections,Infectious Diseases Division,International Centre for Diarrheal Disease Research,Bangladesh(icddr,b); 5 Bangabandhu Sheikh Mujibur Rahaman Agricultural University,Joydebpur,Gazipur-1706,Bangladesh; 6 Plant Genetic Resources Center,Bangladesh Agricultural Research Institute,Joydebpur,Gazipur-1701

**Keywords:** Quantitative trait locus (QTL), Simple Interval Mapping, Composite Interval Mapping, Logarithm-Of-Odds (LOD)

## Abstract

Quantitative trait locus (QTL) analysis is a statistical method that links two types of information such as phenotypic data (trait measurements) and genotypic data (usually molecular markers). There a number of QTL tools have been developed for gene linkage mapping. Standard Interval Mapping (SIM) or Simple Interval Mapping or Interval Mapping (IM), Haley Knott, Extended Haley Knott and Multiple Imputation (IMP) method when the single-QTL is unlinked and Composite Interval Mapping (CIM) is designed to map the genetic linkage for both linked and unlinked genes in the chromosome. Performance of these methods is measured based on calculated LOD score. The QTLs are considered significant above the threshold LOD score 3.0. For backcross-simulated data, the CIM method performs significantly in detecting QTLs compare to other SIM mapping methods. CIM detected three QTLs in chromosome 1 and 4 whereas the other methods were unable to detect any significant marker positions for simulated data. For a real rice dataset, CIM also showed performance considerably in detecting marker positions compared to other four interval mapping methods. CIM finally detected 12 QTL positions while each of the other four SIM methods detected only six positions.

## Background

Phenotypic variations in living creature are observed due to the variation of molecular genetic factor that is called DNA or gene or biomarker. Most of the phenotypes (traits) in organisms are in quantitative in nature [4]. Examples include number of seeds produced in per plant to study the evolutionary fitness, blood pressure to study the hypertension, milk output in dairy breeding etc. [9]. Variation in such quantitative traits is often due to the effects of multiple genetic loci and for environmental factors. In genetics, a QTL is defined as a region of the genome that is associated with an effect on a quantitative trait [1]. A QTL may be a single gene or may be cluster of linked genes that affect the trait. QTL analysis is specialized techniques that construct the genetic linkage maps to locate loci (QTLs) that affect a quantitative trait and estimate the effect of QTLs on the trait [11]. QTL analysis allows researchers in fields as diverse as agriculture, evolution, and medicine to link certain complex phenotypes to specific regions of chromosomes. The goal of QTL analysis is to identify the action, interaction, number, and precise location of these regions [8]. The basic step for mapping QTL includes organizing a cross between two inbred strains differing largely in a quantitative trait: segregating offspring are scored both for the trait and for a number of genetic markers [2]. A cross between two parental inbred lines M1 and M2 is performed to generate an F1 population. The F1 progeny are all heterozygotes with the same genotype. Usually, the segregating progeny are produced by a backcross (B1=F1 x parent) or an intercross (F2=F1 x F1).

Due to modern innovation in molecular biology, it has been easier to make fine-scale genetic maps for a large number of organisms by defining the genomic positions of a number of genetic markers (RFPL, isozymes, RAPDs, AFLP, VNTRs, etc.) and to find a comprehensive classification of marker genotypes by means of dominant markers [2,10]. These rapid expansions of associated techniques in molecular biology have enabled the plant breeders, physiologists, pathologists and other plant scientists to gear up and expedite the detailed genetic mapping and analysis of QTLs. Thoday first introduced the idea of using two markers to bracket a region for testing QTLs [11]. Lander and Botstein carried out a similar, but much upgraded, method to use two adjacent markers to test the presence of a QTL in the interval by performing a Likelihood Ratio Test (LRT) at every position in the interval, which is called Standard Interval Mapping (SIM) or simply Interval Mapping (IM) method [3]. However, SIM can bias identification and estimation of QTLs when multiple QTLs are located in the same linkage group [3,4,5]. Besides, it is also not effective to use only two markers at a time for mapping analysis. To deal with these difficulties, QTL mapping combines SIM with the multiple marker regression analysis studied by Jasen [6], Zeng [12] and this combination is termed as Composite Interval Mapping (CIM). It avoids the use of multiple marker intervals to deal with the problems of mapping multiple QTL by conditioning a test for a QTL on some linked or unlinked markers that diffuse the effects of other potential QTLs.

## Methodology

### Statistical Approaches for QTL Mapping

**Figure 1:**
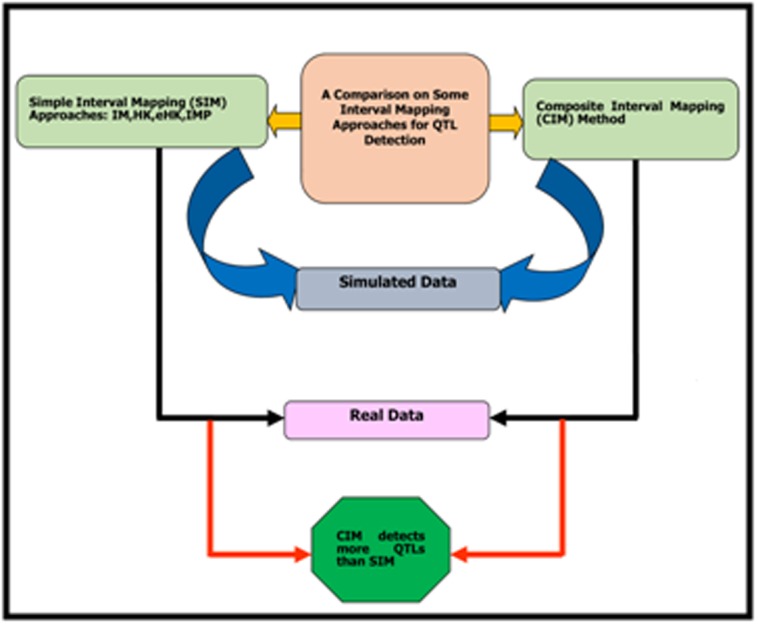
Schematic diagram of this study.

 Analysis of variance (ANOVA) is the basic tool for QTL mapping which is called Marker Regression Method (MR). However, the power of this technique decreases at removal of individuals whose genotypes are missing at the markers and when the markers are widely spaced [8]. There are also a number of statistical methods to overcome this weakness of ANOVA for QTL mapping analysis such as Standard Interval Mapping (SIM) based on maximum likelihood [3], regression based [4] methods are Haley and Knott (HK) , Extended Haley and Knott (eHK), Multiple Imputation methods(IMP). The steps of this study have been briefly demonstrated in Figure 1.

### Simple Interval Mapping (SIM)

Maximum likelihood (ML) and regression based SIM methods are the most popular and widely used interval mapping approaches. These methods make use of a genetic map of the typed markers and like ANOVA, assume the presence of a single QTL. In SIM, each locus is considered one at a time and the logarithm of the odds ratio is calculated for the model that the given locus is a true QTL. The odd ratio is related to the Pearson correlation coefficient between the phenotype and the marker genotype for each individual in the experimental cross. SIM uses two adjacent markers to test the existence of a QTL within the interval by performing a likelihood ratio test (LRT) at every position in the interval [3]. In practice, QTL effects are treated as either fixed or random [16]. In fixed effects QTL model, allelic substitution effects are usually estimated and tested, and QTL, variance is calculated from estimated allelic effects [16]. In random effects QTL model, the QTL effects and QTL variance are directly estimated and tested [3,16]. Since the conditional expectations of the QTL genotype given the flanking marker genotype are unknown in MLE based IM model, this QTL effect model can be treated as a random effects model (REM) [3]. On the other hands, in the HK regression based IM model, the conditional expectation of the QTL genotype given the flanking marker genotype is considered as fixed and this model can be treated as a fixed effect model (FEM) [17].

### Composite Interval Mapping (CIM)

Conventional methods for the detection of quantitative trait locus (QTL) are based on a comparison of single QTL models with a model assuming no QTL. For instance, in the SIM method the likelihood for a single putative QTL is assessed at each location on the genome. However, QTLs located elsewhere on the genome can have an interfering effect. Consequently, the power of detection may be compromised and the estimation of locations and effects of QTLs may be biased [3]. Even non-existing so-called 'ghost' QTLs may appear [4,13]. Therefore, it is obvious that multiple QTLs could be mapped more efficiently and more accurately by using multiple QTL models. One popular approach to handle QTL mapping where multiple QTL contribute to a trait is to iteratively scan the genome and add know QTL to the regression model as QTLs are identified [3]. This method termed as Composite Interval Mapping (CIM) determines both the location and effects size of QTL more accurately than single-QTL approaches especially in small mapping populations where the effect of correlation between genotypes in the mapping population may be problematic [3,18]. CIM performs interval mapping using a subset of marker loci as covariates. These markers function as proxies for other QTLs to increase the resolution of interval mapping by accounting for linked QTLs and reducing the residual variation [18]. In CIM method, suitable marker loci are selected to serve as covariates [3].

### QTL Analysis by SIM and CIM Based on Maximum Likelihood Estimators

Now let us consider no epistasis (QTL x QTL interactions) between QTLs, no intervention (QTL x environmental interactions) in crossing over, and only one QTL in the testing interval. A QTL mapping data includes two parts yj(j = 1,...,n) for the quantitative trait value and Xj(j = 1, ..., n) for the genetic markers and other explanatory variables, for example, gender and food practice. Where yj is the phenotypic value of the Jth individual, Xj is a subset of Xj which may contain some chosen markers and other explanatory variables. To investigate the existence of a QTL at a given position in a marker, we want to test the following statistical hyp1othesis. Null Hypothesis: there is no QTL at a given position vs alternative hypothesis: there is a QTL at a given position.

The principle for QTL mapping is: (a) the Likelihood can be calculated for a given set of parameters (particularly QTL effect and QTL position) given the observed data on phenotypes and marker genotypes. (b) The estimates for the parameters are those where the likelihood are highest. (c) A significance threshold can be established by permutation testing. The number and size of intervals should be considered in determining the threshold value since multiple tests are performed in mapping. The hypothesis is usually tested at every position of an interval and for all intervals of the genome to produce a continuous likelihood ratio test (LRT) statistic profile.

Traditional parametric linkage analysis, commonly known "logarithm (10 base)-of-odds" (LOD score) analysis is based on the likelihood (odds) ratio. This ratio is the relative probability between the probabilities of two alternatives L_HA_/L_H0_, where L_H0_ is the likelihood of no linkage under null hypothesis (recombination fraction is θ=0.5) and L_HA_ is the likelihood under alternative hypothesis of linkage (θ <0.5) developed is a popular statistical tool now widely used by plant breeders in genetics for QTL mapping [14]. The LOD score however compares the likelihood of obtaining the test data if the two loci are actually linked, to the likelihood of observing the same data purely by chance. Positive scores indicate the presence of linkage and the negative scores imply the less likelihood of presence linkage. Computerized LOD score analysis is the simple way to analyze complex family lineages in order to determine the linkage between a trait and a marker or two markers [7].

A LOD score greater than 3.0 is considered evidence for linkage as it indicates 1000 to 1 odds that the linkage being observed did not occur by chance. On the other hand, a LOD score less than -2.0 is considered evidence to exclude linkage [14]. Although it is very unlikely that a LOD score 3 would be obtained from a single pedigree, the mathematical properties of the test allow data from a number of pedigrees to be combined by summing their LOD scores [14]. A LOD of 3 translate to a p-value of approximately 0.05, no multiple testing correction (e.g., Bonferroni correction) is required [14,15].

## Results and Discussion:

### Simulation Study:

To calculate the performance of the SIM/IM, HK, eHK and IMP in comparison of the CIM approach for QTL analysis, we consider backcross population for simulation study. In this comparison, we assume only one QTL on a chromosome with 6 equally spaced markers, where any two successive marker interval size is 1 cM. Marker positions and their genotypes are generated using R/qtl open source software [9] (http://www.qrtl.org/). The successive marker interval size 1 is considered. To generate the simulated data for backcross population we consider the number of individuals (nind=30), number of chromosomes (nchr=4) and number of markers (nmar=6).The true values for the parameters in the SIM model are assumed as a=0.8, µ=0.2.

**Figure 2:**
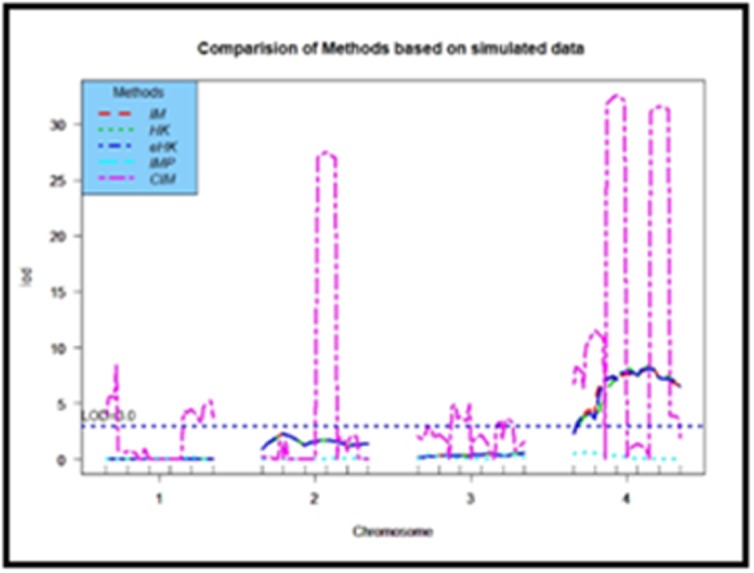
LOD scores curves for comparison of Interval Mapping (IM), Haley-Knott (HK), Extended Haley-Knott (eHK), Multiple Imputation (IMP), and Composite Interval Mapping (CIM) evaluated based on backcross simulated data.

Determination of the performance of the CIM method in comparison of the four methods SIM, HK, eHK is calculated based on LOD score. It is observed from the Figure 2 that for four chromosomes with six markers in each chromosome, the four methods IM, HK, eHK and IMP cannot detect any QTL position by any maker in any position of each chromosome whereas the CIM method identified three QTL positions. One is by the 4th marker in chromosome 2 as well as two positions are detected by the 3rd and 5th markers corresponding to chromosome number 4 whereas the other methods fail to detect any QTLs in each chromosome.

### Comparison Analysis Based on Real Data:

**Figure 3:**
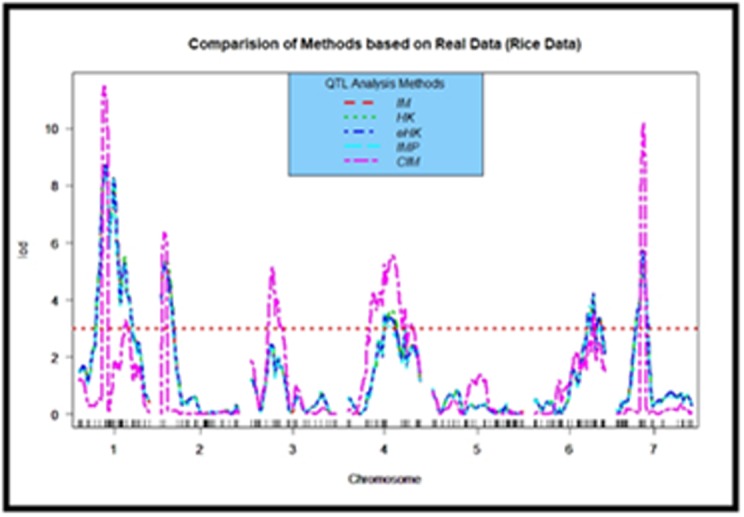
LOD score curves for comparison of Interval Mapping (IM), Haley-Knott (HK), Extended Haley-Knott (eHK), Multiple Imputation (IMP), and Composite Interval Mapping (CIM) evaluated based on real rice mapping population derived from IR64/Azucena.

To investigate the performance of the Composite Interval Mapping (CIM) in comparison of other four simple interval methods for QTL analysis in the scenario of real data, we considered a rice mapping population derived from the parent variety of IR64, an irrigated indica variety and Azucena, a traditional upland japonica variety [9]. The dataset used for QTL analysis consisted of molecular marker data of 200 SSR makers from 7 chromosomes. One phenotypic data such as plant height is taken into consideration of backcross population of 200 recombinant inbred lines (RIL) derived from IR64/Azucena [9]. It was however observed from the Figure 3 that the QTL mapping tool CIM performs better than the other four methods in detecting QTL positions in real dataset. For each chromosome except the chromosome 5 and 6, CIM method detected QTL positions significantly than the other four interval mapping methods.

## Conclusion

The investigation of this comparative study suggests that the Composite Interval Mapping (CIM) performs significantly better than the other four Simple Interval Mapping (SIM) methods in detecting QTL positions in backcross technique both on simulated data and on real dataset. CIM detected three makers in chromosome 2 and 4, as well as other four SIM methods were unable in detecting QTLs for each of the 4 chromosomes for simulated data. In addition, for a real rice data set from backcross population, the CIM performs mostly in similar fashion for detecting QTLs in different positions in each of the 7 chromosomes. CIM were finally able to detect twelve QTLs above the LOD threshold 3.0 whereas other SIM methods identified only six marker positions.
